# Public availability of information from officially accredited medical schools in China

**DOI:** 10.1186/s12909-022-03491-8

**Published:** 2022-05-31

**Authors:** Shaowen Li, Kun Su, Peiwen Li, Yifei Sun, Ying Pan, Weimin Wang, Huixian Cui

**Affiliations:** 1 Medical Education Collaboration and Medical Education Research Center, Hebei Medical University, Shijiazhuang, 050017 China; 2 Foreign Language Education Department, Hebei Medical University, Shijiazhuang, 050017 China; 3 Second Surgery Department, Hebei Provincial Hospital of Chinese Medicine, Shijiazhuang, 050013 China; 4 Law School, University of International Business and Economics, Beijing, 100029 China; 5 Peking University Health Science Center, Beijing, 100191 China

**Keywords:** Public availability of information, Accredited medical schools, WFME Global Standards, Medical education accreditation, Transparency, Stakeholders, Accreditation results

## Abstract

**Background:**

Medical education accreditation in China has been conducted by the Working Committee for the Accreditation of Medical Education (WCAME) and 129 medical schools have completed accreditation by December 2021. Despite studies on the standards, process and effectiveness of accreditation, the actual information transparency of accredited medical schools in China has not been examined. The study investigated the status of publicly available information from WCAME-accredited medical schools in China, and whether public availability of information had significant differences among different types of universities.

**Methods:**

The 129 medical schools’ official websites were reviewed for the 21 criteria of the *WFME Global Standards for Quality Improvement: Basic Medical Education*. Dichotomous method was used to record information as presence or absence. SPSS was utilized for descriptive and ANOVA analyses.

**Results:**

The mean of the publicly available information on the 21 criteria was 13.77 ± 3.57, and only 5 (3.9%) accredited medical schools had all relevant information available. Publicly available information on Governance (100%) and Administration (100%) was the most, whereas information on Assessment in support of learning (16.3%) was the least. Public availability of information differed significantly among schools accredited with higher (18.15 ± 2.16), medium (13.69 ± 3.41) and lower results (12.79 ± 3.19) (*F* = 14.71, *p* < 0.05). Medical universities and comprehensive universities did not show significant differences in their overall information availability (*F* = 0.25, *p* > 0.05). Central government funded universities had a remarkably larger amount of publicly available information than local government funded universities (17.86 ± 1.98 vs. 12.75 ± 2.93, *p* < 0.05).

**Conclusion:**

Public availability of information from the accredited medical schools in China needs to be improved to promote transparency and continuous quality improvement, especially with regard to information on curriculum, assessment and quality assurance. Explicit information availability requirements need to be considered to include in medical education standards, and further studies are warranted to explore which information elements should be made publicly available.

## Introduction

Accreditation of medical schools and medical programs has been adopted worldwide as a quality assurance process. Medical education accreditation is defined as an external quality evaluation mechanism where a medical educational institution or a medical training program is reviewed by a designated authority using a set of publicized standards and predetermined protocols [1–4]. The main purposes are to evaluate whether the medical education program meets the basic quality standards, to encourage medical schools to perform continuous quality improvement, and to improve the quality of medical graduates [2, 5]. Since heterogeneity existed in the performance of medical education accreditation across countries and regions [3, 6, 7], studies are needed to understand medical education accreditation in different countries.

According to data from Directory of Chinese Medical Schools, China, with a total of 445 higher education institutions providing medical education, and 70480 newly enrolled medical students in the year 2020 [8], is indeed a big country for medical education. In China, medical education includes 11 broad categories, including basic medicine, clinical medicine, stomatology, public health and preventive medicine, traditional Chinese medicine, etc., and clinical medicine is the main body of the medical education system in China, with 192 medical schools providing clinical medicine education. China has a history of clinical medicine education accreditation for over a decade, which is conducted by the Working Committee for the Accreditation of Medical Education (WCAME) of China. WCAME is a specialized organization established by the Ministry of Education to organize and conduct clinical medicine professional accreditation and is the only national organization active in this field. WCAME devised its accreditation standards based on and in compliance with the global standards of basic medical education published by the World Federation for Medical Education (WFME). In this regard, WFME has a huge influence on the clinical medicine education accreditation in China. In June 2020, WCAME was officially recognized by WFME, which marked a significant milestone for medical education accreditation in China, fostered the international recognition of Chinese qualifications in the domain of medicine, and signified the establishment of a medical education accreditation system with Chinese characteristics and international equivalence.

However, as is required by the Educational Commission for Foreign Medical Graduates (ECFMG), certification of international medical graduates will be limited to graduates of medical schools accredited by authorities recognized by WFME before the deadline of 2023 [9]. Until December 2021, WCAME has completed the accreditation process for 129 medical schools in China, and the standards they use derive from an adaptation of WFME standards. In 2020, the WFME published an updated edition of *the WFME Global Standards for Quality Improvement: Basic Medical Education* (the 2020 WFME Standards), and the WCAME is currently planning the revision of *Accreditation Standards for Basic Medical Education*in China based on the 2020 WFME Standards. Applying the 2020 WFME Standards to Chinese medical schools to evaluate the status of publicly available information may provide useful data for WCAME’s revision of standards (to be used for the new round of accreditation), since it is important to take both international standards and local context into account when setting medical education standards [10]. Besides, the 2020 WFME Standards can be used as a guideline to provide an overall picture of medical schools’ public availability of information, and as a yardstick against which to examine what aspects medical schools are doing well and what areas need improvement.

The 2020 WFME Standards emphasized transparency by making explicit requirements on certain information to be “publicly available”. In Standard 1.1 Stating the mission, for example, the document clearly stated that “The school has a public statement that sets out its values, priorities, and goals” [11]. And in Standard 3.1 Assessment policy and system, a statement specifying “The school has a policy that describes its assessment practices. The policy is shared with all stakeholders” [11] is clearly stated. Baniadam and colleagues have examined the public availability of information from WFME-recognized accreditation agencies as a marker of their transparency [12]. Information transparency is becoming increasingly important, as commitments are made to continuously enhance quality of medical education in addition to the challenges created by globalization [13]. Education transparency refers to the situation where information of educational institutions and decisions made in the institutions are shared in a clear, understandable and accessible manner to the stakeholders in a way that is consistent with relevant requirements and security issues. Previous studies have investigated admission transparency, external examiner transparency, school supervision transparency, donor sponsorship transparency, etc., raised the importance of higher education transparency. Despite wide emphasis on university transparency and WFME’s appeal to make information publicly available, medical schools’ information availability has not been emphasized, and the actual information transparency from officially accredited medical schools in China has not been examined.

Therefore, the study was conducted to use the 2020 WFME Standards as a guideline to evaluate the publicly available information from all the accredited medical schools in China. Since medical education involves high costs, numerous resources, and long duration, promoting medical schools’ publicly available information is of great significance to all its stakeholders. Specifically, this study aimed to evaluate the status of publicly available information from WCAME-accredited medical schools in China; and to examine whether different types of universities had significant differences in the amount of publicly available information. Therefore, our research questions were to examine the amount of publicly available information and compare the total amount between: (1) medical universities and comprehensive universities; (2) universities funded by the central government and those funded by the local government; and (3) universities with a higher, medium and lower past (i.e., pre-WCAME recognition) accreditation result.

## Methods

### Definition of publicly available information

The 2020 WFME Standards was employed as a guideline to examine the public availability of information from Chinese medical schools, as WFME Standards have been endorsed and employed worldwide as templates for the establishment of national and regional accreditation standards [2, 6], and have been widely accepted as bases for improving medical education and proved to be easily adaptable to national and local contexts [14–16]. In the study, information was viewed as “publicly available” if it were accessible by anyone seeking it on the schools’ websites, and if the information missing on the websites could be provided via request within 7 working days.

### Officially accredited medical schools in China

Initially started in 2006, the WCAME has accredited 129 universities providing medical education as of December 1^st^, 2021. The vast majority (127) of the 129 universities were funded publicly (either by the central or the local government), and only two schools were privately funded, which were not included in part of the data analysis when the central government and local government funded universities were compared. To be noted, Harbin Medical University is first medical school being accredited by WCAME and is the only university that has completed two rounds of certification. In the present study, we adopted the first round of certification result as the recorded data for Harbin Medical University, as the rest of the schools had all completed only the first round of accreditation. The full list of accredited medical schools and their accreditation results (accreditation approval length in years) was obtained from WCAME website and double checked with the Secretary of WCAME.

### Research methods

From September 1^st^ to December 1^st^ in 2021, 2 researchers (R1 and R2) independently reviewed each of the universities’ official websites (Chinese web pages) for the 21 information elements in the 2020 WFME Standards. The public availability of information was recorded in a dichotomous method, with information elements recorded as present or absent. For information elements recorded as “present”, they should either be obtained as website texts or downloadable and unencrypted documents, whose link should be recorded in case of further discussion. If an information element is otherwise not present, it should be recorded as absent. Researchers R1 and R2 independently reviewed the websites and rated relevant information in a dichotomous cutoff, i.e., present (1) or absent (0), which would improve the reliability of the results compared with making judgments on the quality of information. The two researchers met to check their records of information availability, and were joined by a third researcher (R3) for any discrepancies. After the results achieved consistency, for those information elements that were unavailable after checking every web pages until no clickable content was available, researcher R3 attempted to contact the institution through telephone or email, clarified requests on inaccessible information and waited for the provision of corresponding information for 7 working days.

### Study outcomes

The primary outcome was the accredited medical schools’ publicly available information elements. The secondary outcomes included each medical school’s accreditation approval length (in years), as obtained from the WCAME website; and types of the accredited medical institutions, which were obtained from the university websites and double checked with the Ministry of Education website.

According to the *WCAME’s Guidelines for Accreditation*, WCAME reviews the assessment team members’ recommended accreditation decision and makes the final decision on accreditation as either accredited or non-accredited [17]. For accredited institutions, the WCAME specified a validity period of accreditation, effective from the end of the site visit [17]. In the study, the accreditation approval length (4–8 years) were collected for all WCAME-accredited universities, and were classified into three groups: lower accreditation result group (4–5 years), medium accreditation result group (6–7 years), and higher accreditation result group (8 years).

We utilized two ways to categorize the accredited medical schools: (a) whether the university was a medical university or a comprehensive university; and (b) whether the university was financed by the central government or by the local government. The first categorization was adopted considering the long and wide discussions in China on whether an independent medical university or a comprehensive university can provide higher quality medical education. Medical universities were defined as higher education institutions providing at least one medical education program, and providing medical education programs exclusively. The medical education programs include basic medicine, clinical medicine, stomatology, public health and preventive medicine, traditional Chinese medicine, Chinese medicinal herb, integrative medicine, pharmacy, medical technology, forensic science, and nursing. Comprehensive universities were defined as higher education institutions constituted by a variety of colleges and departments, with a medical college included.

In the second categorization, central government funded and local government funded universities were compared to explore whether there were statistical differences in their publicly available information, considering they were all public universities but with different sources of funding, and that central government funded universities were generally reputed to have higher education quality.

### Data analysis and ethical approval

Descriptive statistical analysis was employed to calculate the sums of publicly available information elements for each school, the numbers and frequencies of available and absent information elements, the mean of the 21 publicly available information elements, and the mean accreditation approval length for all the accredited schools. We then categorized schools with a lower, medium, and higher accreditation approval length into three groups to examine whether they showed any statistically significant differences in public availability of information. ANOVA analyses with descriptive analysis, homogeneity tests, and Post-hoc comparisons were performed, with the sums of publicly available information elements as dependent variable and three groups of accreditation results as independent variable to test the information availability differences among schools a higher, medium and lower accreditation result. We then performed an ANOVA analysis with the sums of publicly available information elements as dependent variable and type of university (medical vs. comprehensive) as independent variable to evaluate whether medical universities and comprehensive universities showed any significant differences in their public availability of information. Same analysis was conducted with type of funding (central vs. local) as independent variable to test the differences in publicly available information from central government funded and local government funded universities. The significance level (*p* value) for all statistical tests was 0.05. Statistical analysis was performed with the use of SPSS version 25.0. The study protocol was reviewed and approved by Medical Ethics Committee of Hebei Medical University and deemed non-human subjects research.

## Results

### Accredited medical schools and characteristics

According to Directory of Chinese Medical Schools, there are a total of 192 higher education institutions in China providing clinical medicine education. The clinical medicine training programs currently include 5-year undergraduate clinical medicine program, "5 + 3" training system of master of clinical medicine, and 8-year M.D. clinical medicine program. Among the 192 medical schools providing clinical medicine programs, 14 schools offer the 8-year program, and 27 schools offer the "5 + 3" program. Medical training in China is mainly offered through 5-year undergraduate programs, aiming to produce sufficient qualified medical professionals to meet the vast and increasing health-care needs.

Until December 2021, a total of 129 medical schools in China have been officially accredited by the WCAME. The 129 accredited medical schools cover 25 provinces and 4 municipalities (Beijing, Tianjin, Shanghai, Chongqing) in China and they are all urban universities. Specifically, 9 (6.98%) universities are located in municipalities, 53 (41.09%) are in provincial capital cities, and 67 (51.94%) are in non-provincial capital cities.

In terms of the types of the accredited institutions, 59 (45.7%) are medical universities, and 70 (54.3%) are comprehensive universities offering clinical medicine programs. 22 (17.1%) are central government funded universities, and 105 (81.4%) are sponsored by the local government.

With regard to medical schools’ accreditation results, the mean accreditation approval length was 5.87 ± 0.99 years for the 129 medical schools. After categorization into three groups, we found there were 13 (10.1%) medical schools which obtained a relatively higher accreditation result (8 accredited years), 71 (55.0%) received medium accreditation results (6–7 accredited years) and 45 (34.9%) received a relatively lower result (4–5 accredited years).

### General status of public availability of information

Of the 129 officially accredited medical schools, only 5 (3.9%) universities had all the 21 information elements publicly available. There were 5 (3.9%) schools providing publicly available information on 20 criteria, 4 (3.1%) schools providing information on 19 criteria, 8 (6.2%) schools providing information on 18 criteria, and 7 (5.4%) schools with publicly available information on 17 criteria. Across all the accredited medical schools, the mean of publicly available information elements was 13.77 ± 3.57. The sums of the accredited medical schools’ available information ranged from 6 to 21. There was 1 university providing only 6 information elements on its website, which was equivalent to a minor 28.6% availability rate. And there were 2 universities providing only 7 elements of information, which also seemed unsatisfactory.

### Public availability of information according to the 2020 WFME standards

The status of publicly available information elements according to the 2020 WFME Standards were shown in Table 1. Publicly available information on Governance (100%) and Administration (100%) was the most, whereas publicly available information on Assessment in support of learning (16.3%) was the least.

**Table 1:** Information availability according to the 21 criteria

**Criteria**	**Number (percentage) of present information**	**Number (percentage) of absent information**
Std 8.1 Governance	129 (100%)	0
Std 8.3 Administration	129 (100%)	0
Std 1.1 Stating the mission	128 (99.2%)	1 (0.8%)
Std 4.1 Selection and admission policy	127 (98.4%)	2 (1.6%)
Std 5.1 Academic staff establishment policy	127 (98.4%)	2 (1.6%)
Std 6.1 Physical facilities for teaching and learning	126 (97.7%)	3 (2.3%)
Std 5.2 Academic staff performance and conduct	117 (90.7%)	12 (9.3%)
Std 6.2 Clinical training resources	114 (88.4%)	15 (11.6%)
Std 4.2 Student counselling and support	102 (79.1%)	27 (20.9%)
Std 6.3 Information resources	91 (70.5%)	38 (29.5%)
Std 3.4 Quality control	89 (69.0%)	40 (31.0%)
Std 7.1 The quality assurance system	74 (57.4%)	55 (42.6%)
Std 2.1 Intended curriculum outcomes	72 (55.8%)	57 (44.2%)
Std 5.3 Continuing professional development for academic staff	67 (51.9%)	62 (48.1%)
Std 8.2 Student and academic staff representation	54 (41.9%)	75 (58.1%)
Std 2.2 Curriculum organization and structure	53 (41.1%)	76 (58.9%)
Std 2.3 Curriculum content	49 (38.0%)	80 (62.0%)
Std 3.3 Assessment in support of decision-making	45 (34.9%)	84 (65.1%)
Std 2.4 Educational methods and experiences	37 (28.7%)	92 (71.3%)
Std 3.1 Assessment policy and system	25 (19.4%)	104 (80.6%)
Std 3.2 Assessment in support of learning	21 (16.3%)	108 (83.7%)

For the missing information according to the 2020 WFME Standards, researcher R3 made attempts to contact the universities via phone or email as were listed on universities’ websites. However, 126 contacts were attempted to make, with only 4 universities providing the information we requested. Common reasons for the denial of providing adequate information included: invalid telephone numbers (*n* = 9), no one answered the phone (*n* = 87), claiming all the information was publicly available online (*n* = 23), confidentiality (*n* = 57), referral to other departments (*n* = 65), no permission from superiors (*n* = 23), and no reply of emails (*n* = 119).

### Public availability of information and accreditation results

The means of publicly available information elements were 12.79 ± 3.19, 13.69 ± 3.41 and 18.15 ± 2.16, respectively, for medical schools obtained a lower, medium, and higher accreditation approval result. And results from the ANOVA analysis showed that there were statistically significant differences in the public availability of information among medical schools accredited with higher, medium and lower results (*F* = 14.71, *p* < 0.05). Medical schools in the higher accreditation approval length group had more publicly available information elements than those in the medium and lower accreditation length groups; and medical schools accredited with medium results had more publicly available information elements than those with lower results.

### Public availability of information and types of schools

Of the 129 accredited medical schools, 59 (45.7%) were medical universities and 70 (54.3%) were comprehensive universities. The means of publicly available information elements were 13.39 ± 3.65 for medical universities and 14.09 ± 3.50 for comprehensive universities. Results from the ANOVA analysis showed that there was no statistically significant difference in the public availability of information from medical and comprehensive universities (*F* = 0.25, *p* > 0.05).

Results from the ANOVA analysis showed central government funded universities and local government funded universities differed significantly in their overall status of public availability of information (*F* = 46.50, *p* < 0.05). Central government funded universities had a remarkably larger amount of publicly available information elements than their regional government funded counterparts (17.86 ± 1.98 as compared with 12.75 ± 2.93).

## Discussion

### General public availability of information from the 129 medical schools

The study examined 129 WCAME-recognized universities’ transparency by reviewing universities’ websites for 21 criteria in the 2020 WFME Standards. Results of the study showed the relatively unsatisfactory overall status of public availability of information from medical schools in China. The finding is consistent with Baniadam and colleagues’ study of 20 WFME-recognized agencies’ transparency, which revealed that while 12 agencies had over 90% of expected information elements related to accreditation standards, procedures, and processes available on agency websites, 6 agencies had less than 50% information elements accessible [12]. They indicated that some WFME-recognized agencies present significant barriers for stakeholders of accreditation due to a lack of transparency [12]. Our result echoed Verkijika and De Wet’s finding in their accessibility evaluation of all 26 South African university websites that none of the websites met all the WCAG (Web Content Accessibility Guidelines) 2.0 accessibility criteria [18]. Our finding was also in consistency with the systematic literature review that the university websites of 9140 universities in 67 countries violated most of the accessibility guidelines and presented important accessibility problems [19]. The generally lower public availability of information in the accredited medical schools in China clearly highlighted the need for universities and colleges providing clinical medicine education to make more relevant information available to stakeholders. Public availability of information on medical schools’ websites is of particular interest to its stakeholders, including potential medical students, parents, policy makers, patients, future employers, higher education regulation agencies, and medical education quality assurance agencies, etc. Transparency of information can provide essential knowledge for key stakeholders, permit stakeholders’ awareness and engagement in medical education, enable informed decisions, promote continuous quality improvement, and optimize medical education quality assurance. As Barzansky et al. has emphasized, medical schools should review their compliance with accreditation standards internally and regularly, to facilitate the establishment of a continuous quality improvement culture in medical schools [20].

With insufficient information provided on the universities’ Chinese web pages, there is a likelihood that publicly available information in English on the websites may be even less. Globalization has been prevalent in medical education as recent years have seen an increasing number of cross-border health professionals’ movements and international students pursuing medical programs overseas [21, 22]. Many universities in China now provide parallel undergraduate medical courses in English for international students [23]. However, not all medical schools in China provide an official English website, and information available in English tend to be far less than that in Chinese. Studies are needed to systematically retrieve information on universities’ English official web pages to evaluate the publicly available information in English, promoting Chinese universities to improve their availability to international stakeholders, who tend to have a limited Mandarin level [23]. 2023 marked the deadline to meet the ECFMG’s requirement for certification of international medical graduates to graduates of medical schools accredited by WFME-recognized authorities [9]. Accreditation of medical schools and programs according to established standards helps to safeguard the immigration of healthcare professionals, the practice of medicine and the employment of medical workforce. And trends are becoming increasingly prevalent for medical professionals to study and work globally [21, 24, 25]. In this regard, public availability of information both in Chinese and English should be improved for the benefits of both domestic and overseas students.

The study found medical schools in China rarely responded efficiently and effectively on information requests. While it may be reasonable to assume that universities had related information and decided not to publish certain information elements on their websites, the fact that we gained fruitless results upon further requests of missing information is quite concerning. The limited information publicly available on the accredited medical schools’ official websites, lack of response to requests of information or various ways of refusal to provide further information to researchers, brought challenging barriers to stakeholders’ knowledge of medical education quality.

### Public availability with regard to the 21 criteria

We calculated the information availability of the 129 medical schools with regard to the 21 criteria, and found the less frequently available information elements were closely related to assessment and curriculum, which might be provided to students currently enrolled in the courses or programs via intranet or course materials, instead of being made publicly available on universities’ official websites. It is also likely that those information elements were less accessible because their accessibility was relatively less important and relevant. Reasons for universities’ decisions on what to publish on their websites should be understood to shed light on the revision of medical education standards in China. China is one of the countries seeking to align accreditation practices with global standards, and the WCAME used external standards as a template and revised them to set the standards for clinical medicine education in China. In addition to consistency with international demands, it has been emphasized that countries’ “glocalization” of medical accreditation standards should give adequate consideration to local values and societal needs [10]. Therefore, providing rationales for requiring certain information elements to be publicly available, and understanding universities’ mechanism on what information elements to be published on their websites are necessary.

Though standard setting authorities should consider the relative importance and relevance for requiring those areas of information to be shared with stakeholders, medical schools should raise their consciousness and emphasis concerning transparency on assessment, curriculum, and quality assurance. With important information about curriculum, assessment and quality assurance commonly unavailable, exchanges of experience and good practice among medical schools would be hindered and knowledge about medical schools’ quality would be inconveniently obtained.

### Public availability and accreditation results

Findings from the study indicated that medical schools accredited with different levels of results showed statistically significant differences in their status of public availability of information. The schools which obtained higher level of accreditation conclusions tended to have more publicly available information. This may be explained by the possibility that universities granted a higher accreditation result had a better overall performance. Currently, WCAME’s accreditation procedures include medical schools’ submission of self-assessment report, expert team’s site visit, working committee’s revision of accreditation report and recommendations, final accreditation report and final decision on accreditation, etc. The study highlighted the need for medical schools’ public availability of information to be considered and evaluated as part of the accreditation process, to promote medical schools’ education transparency and engagement of key stakeholders.

### Public availability and types of schools

The study found that medical and comprehensive universities did not show any statistically significant differences in their information availability. It has long been debated in China about whether medical or comprehensive universities were superior in training health professionals, and since the founding of People’s Republic of China, major trends for providing medical education in medical universities and for providing medical education in comprehensive universities have altered for several times. Findings from the study may resolve some of the debates by contributing that medical and comprehensive universities did not differ in information transparency.

The study found universities funded by the central government had a significantly higher overall information availability rate than those funded by local government. This may derive from the fact that the central government funded universities were generally having a longer history, embracing more financial and educational resources and support, and having more excellent academic performance and better student enrollment than most locally funded universities. Our findings were consistent with Yuan and Chen’s [26] comparison of the financial performance of universities financed by the central government and those by the local government, which revealed the universities financed by the central government had a better outcome. Numerous researchers have noticed the unbalanced development of central government funded universities and local government funded universities, among which, Wang and Zhang compared the number of universities of each type, and found the scale of local higher education institutions was expanding rapidly, whereas the number of universities financed by the state central government remained unchanged throughout the decades, leading to local universities’ fierce competition of limited educational and financial resources [27].

### Limitations

Our study has some potential limitations. First, we only evaluated the current status of public availability of information. Further research is warranted to examine the reasons for universities’ decisions about what to publish on their websites. Qualitative methods may help to uncover valid reasons for discrepancies not captured by the methods of this study. Another limitation is that we retrieved and reviewed information from the Chinese web pages of the accredited schools, and studies are needed to evaluate public availability of information on the English web pages, considering globalization of medical education and the increasing number of international stakeholders. Plus, we collected data by searching the official websites throughout 3 months’ duration, and public availability of information may change due to the dynamic nature of online information. And studies are needed to investigate how well schools performed in the initial site visit, and how things have changed from then on.

## Conclusion

Accreditation of medical schools has become a common practice for most parts of the world to promote assurance to the public that medical schools are providing medical education of high quality. Although the standards, procedures and effectiveness of accreditation of medical education have been studied, to the best of our knowledge, this is the first study to examine the public availability of information in all the WCAME-accredited medical schools in China. The overall public availability of information from the accredited medical schools in China needs to be improved. Universities accredited with a longer accreditation approval length revealed a significantly larger amount of publicly available information. Though medical and comprehensive universities did not differ significantly in their public availability of information, universities funded by the central government had a significantly larger amount of publicly available information than those funded by the local government. Besides emphasis on improving medical schools’ transparency, standards requiring certain information elements to be made publicly available are open to discussion, and rationales for universities’ decisions on what to publish on their websites need to be further understood. It is also suggested that for the WCAME’s upcoming revision of its standards, both international standards and local context need to considered, and criteria on public availability of information need to be taken into account.

## Data Availability

The datasets analyzed in the current study are available from the corresponding author on reasonable request.

## Abbreviations

WCAME: Working Committee for the Accreditation of Medical Education
WFME: World Federation for Medical Education
2020 WFME Standards: The WFME Global Standards for Quality Improvement: Basic Medical Education
ECFMG: Educational Commission for Foreign Medical Graduates
